# The Bacterial Flora Associated with the Polyphagous Aphid *Aphis gossypii* Glover (Hemiptera: Aphididae) Is Strongly Affected by Host Plants

**DOI:** 10.1007/s00248-019-01435-2

**Published:** 2019-12-04

**Authors:** Shifen Xu, Liyun Jiang, Gexia Qiao, Jing Chen

**Affiliations:** 1 Key Laboratory of Zoological Systematics and Evolution, Institute of Zoology, Chinese Academy of Sciences, Beijing, 100101 China; 2 College of Life Sciences, University of Chinese Academy of Sciences, Beijing, 100049 China

**Keywords:** Polyphagous species, *Arsenophonus*, Host specialization, Symbiont–symbiont interactions

## Abstract

Aphids live in symbiosis with a variety of bacteria, including the obligate symbiont *Buchnera aphidicola* and diverse facultative symbionts. The symbiotic associations for one aphid species, especially for polyphagous species, often differ across populations. In the present study, by using high-throughput 16S rRNA sequencing, we surveyed in detail the microbiota in natural populations of the cotton aphid *Aphis gossypii* in China and assessed differences in bacterial diversity with respect to host plant and geography. The microbial community of *A. gossypii* was dominated by a few heritable symbionts. *Arsenophonus* was the most dominant secondary symbiont, and *Spiroplasma* was detected for the first time. Statistical tests and ordination analyses showed that host plants rather than geography seemed to have shaped the associated symbiont composition. Special symbiont communities inhabited the Cucurbitaceae-feeding populations, which supported the ecological specialization of *A. gossypii* on cucurbits from the viewpoint of symbiotic bacteria. Correlation analysis suggested antagonistic interactions between *Buchnera* and coexisting secondary symbionts and more complicated interactions between different secondary symbionts. Our findings lend further support to an important role of the host plant in structuring symbiont communities of polyphagous aphids and will improve our understanding of the interactions among phytophagous insects, symbionts, and environments.

**Electronic supplementary material:**

The online version of this article (10.1007/s00248-019-01435-2) contains supplementary material, which is available to authorized users.

## Introduction

Aphids are well known for their symbiotic associations with bacteria. Almost all aphid species harbor the primary endosymbiont *Buchnera aphidicola*, which inhabits specialized bacteriocytes and provides aphids with important nutrients for their growth and reproduction [1–4]. *Buchnera* is strictly maternally inherited [5, 6] and has undergone parallel diversification with its aphid hosts [7–14].

Aphids also host multiple secondary (or facultative) bacterial symbionts that are generally not essential for their survival or reproduction. Some are commonly studied, such as *Arsenophonus*, *Fukatsuia symbiotica*, *Hamiltonella defensa*, *Regiella insecticola*, *Rickettsiella viridis*, and *Serratia symbiotica* from the Gammaproteobacteria; *Rickettsia* and *Wolbachia* from the Alphaproteobacteria; and *Spiroplasma* from the Mollicutes [15–21]. These secondary symbionts reside in bacteriocytes, sheath cells, or hemocoel [22] and are transmitted maternally and horizontally [18, 23, 24]. They have environmentally dependent effects on host aphids, including increasing heat tolerance [25–29], protecting against parasitic wasps [30–34] and fungal pathogens [29, 35–37], influencing aphid fitness on host plants [38–40], modifying body color [41], and affecting aphid reproduction [42, 43]. More details are reviewed in Oliver et al. [44], Zytynska and Weisser [45], and Guo et al. [46]. In addition, several facultative symbionts seem to have established co-obligate associations along with *Buchnera* in certain aphid species, such as *Serratia symbiotica*, *Erwinia haradaeae*, *Fukatsuia symbiotica*, *Hamiltonella defensa*, and *Sodalis* in some Lachninae species [21, 47–51] and *Wolbachia* in *Pentalonia nigronervosa* [52, 53].

The associations between microbial symbionts and aphids are quite different in different aphid species. Symbionts carried by one aphid species also often vary across populations. There seems to be a widespread pattern in polyphagous aphids that the populations feeding on different host plants differ in their symbiont communities [54–56]. Most studies have focused on the pea aphid *Acyrthosiphon pisum*, which consists of at least eleven biotypes adapted to specific host plants [57]. Facultative symbionts in the pea aphid exhibit large variation across plant-adapted populations in terms of composition and prevalence [35, 54, 58–61]. Links between particular symbionts and plants have been observed, such as associations between *Hamiltonella defensa* and alfalfa and *Regiella insecticola* and clover. A nonrandom distribution of bacterial symbionts across host plants has also been reported in other polyphagous aphid species such as *Aphis craccivora* [55, 62] and the oligophagous aphids *Phylloxera notabilis* [63] and *Aphis citricidus* [64]. Nevertheless, several studies have highlighted the role of geography in structuring the community of aphids’ bacterial partners. Tsuchida et al. [65] revealed characteristic geographical distribution patterns of secondary symbionts that infected *Acyrthosiphon pisum* in Japan, particularly for *Regiella*. Jones et al. [66] found that the symbiont communities of *Aphis gossypii* and *Pentalonia caladii* varied across aphid populations from different Hawaiian islands. Some studies also indicated correlations between aphid symbionts and other factors, including developmental stage of aphids [67, 68], rearing condition [69], plant species richness [70], and season [71].

The cotton aphid, *Aphis gossypii* Glover, is a cosmopolitan insect pest causing serious economic losses in agriculture. It feeds on many important crops, including cotton, cucurbits, citrus, eggplant, peppers, potato, and flowering ornamental plants such as *Hibiscus* [72]. Several studies have been conducted on bacterial communities. Najar-Rodríguez et al. [73] and Jones et al. [66] investigated the microbial diversity of natural aphid populations from Japan and Australia and from Hawaii, respectively, and highlighted the effect of geography on bacterial profiles. Zhao et al. [74] also found distinct bacterial community structures from different geographic populations feeding on *Bt* cotton in northern China. *A. gossypii* is currently controlled primarily by insecticides, which have been reported to influence the bacterial communities associated with aphids [75, 76]. In addition, by utilizing quantitative PCR, Ayoubi et al. [77] uncovered development-associated dynamics in the abundance of symbionts within *A. gossypii*.

Although geography has been proposed to have a role in structuring the bacterial communities of *A. gossypii*, samples used in previous studies were restricted to a few plants. A detailed and deep exploration of the microbiota in natural populations of *A. gossypii* is still lacking. In this study, using Illumina sequencing of 16S rRNA gene, we characterized the microbial communities of *A. gossypii* collected from diverse plants and different regions in China, assessed differences in bacterial community according to host plant and geography, and discussed the interactions between symbionts.

## Material and Methods

### Sample Collection and DNA Extraction

A total of 110 samples of *Aphis gossypii* feeding on plants belonging to 25 families were collected from 23 regions of China (Table S1). Specimens from the same colony were preserved in 75% and 100% ethanol for making voucher slides and DNA extraction, respectively. The slide-mounted specimens were identified based on the external morphology. All voucher specimens and samples were deposited in the National Zoological Museum of China, Institute of Zoology, Chinese Academy of Sciences, Beijing, China.

A single adult was chosen from each sample for DNA extraction. To remove microbial contaminants from the body surface, each aphid individual was washed with 70% ethanol for 5 min and then rinsed with sterile ultrapure water once for 5 min and four times for 1 min. DNA was extracted from the whole body of a single individual using DNeasy Blood & Tissue Kit (QIAGEN, Hilden, Germany) following the manufacturer’s protocol. A blank sample of sterile ultrapure water was also processed through the same extraction protocol to serve as a negative control during the DNA extraction. The standard cytochrome oxidase subunit I (COI) barcodes were amplified by universal primers (LCO1490: 5′-GGTCAACAAATCATAAAGATATTGG-3′; HCO2198: 5′-TAAACTTCAGGGTGACCAAAAAATCA-3′) [78] to test the quality of DNA extracts, to verify the aphid species identification, and to detect contamination from parasitoid wasps.

### PCR Amplification, Library Preparation, and Sequencing

DNA was amplified using the universal primers of the V3–V4 region of 16S rRNA gene (338F: 5′-ACTCCTACGGGAGGCAGCA-3′; 806R: 5′-GGACTACHVGGGTWTCTAAT-3′). The first polymerase chain reaction (PCR) was carried out in a 50-μL volume containing 1.5 μL (10 μM) of each primer, 0.4 U Q5 High-Fidelity DNA Polymerase (New England Biolabs, Ipswich, MA, USA), 10 μL 5× Q5 Reaction Buffer (New England Biolabs), 10 μL 5× Q5 High GC Enhancer (New England Biolabs), 1 μL dNTPs (New England Biolabs), and 40–60 ng DNA extract. The reaction conditions were as follows: initial denaturation at 95 °C for 5 min, followed by 15 cycles of 95 °C for 1 min, 50 °C for 1 min, 72 °C for 1 min, and final elongation at 72 °C for 7 min. The PCR products were purified using VAHTS™ DNA Clean Beads (Vazyme Biotech, Nanjing, China). In the next step, 10 μL of the purified product was ligated to adapter and sample barcode in a 40-μL volume containing 1 μL (10 μM) of each fusion primer and 20 μL of 2× Phusion High-Fidelity PCR Master Mix (New England Biolabs). The second PCR conditions were as follows: 98 °C for 30 s, 10 cycles of 98 °C for 10 s, 65 °C for 30 s, and 72 °C for 30 s, followed by a final extension at 72 °C for 5 min. Negative amplification controls (sterile ultrapure water) were also included in all PCR reactions. The final PCR products were recovered using 1.8% agarose gel electrophoresis, purified with VAHTS™ DNA Clean Beads (Vazyme Biotech) and then quantified by NanoDrop 2000 (Thermo Scientific, Wilmington, DE, USA). All positive PCR products were mixed at a mass ratio of 1:1. Finally, the library pool was submitted to an Illumina HiSeq 2500 platform (Illumina, San Diego, CA, USA) for paired-end sequencing. The raw reads have been deposited in the NCBI Sequence Read Archive (SRA) database under BioProject accession number PRJNA543947.

### Sequence Processing and Analyses

Paired-end reads were assembled using FLASH v1.2.11 [79]. The merged tags with an average quality score lower than 20 in a 50-bp sliding window were trimmed using Trimmomatic v0.33 [80]. The remaining tags shorter than 300 bp were also removed. High-quality clean tags were then obtained after removing chimeras with UCHIME v8.1 [81]. The denoised sequences were clustered into operational taxonomic units (OTUs) at 97% sequence similarity by the UCLUST module from QIIME [82]. Taxonomy was assigned to all OTUs by searching against the Silva databases [83] using the RDP classifier within QIIME [84]. The OTUs were then filtered with a threshold value of 0.005% of all sequences [85], except for the OTUs that could be classified. Finally, an OTU table containing the number of sequences per sample and taxonomic information was generated.

### Statistical Analyses

Alpha diversity indices (i.e., Shannon and Simpson diversity indices) for each sample were calculated using the *diversity* function in the R package *vegan* [86]. The relative abundance of each bacterial genus was estimated by normalizing the number of sequences assigned to each genus against the total number of sequences obtained for a given sample using the *decostand* function of *vegan*. To better investigate the symbiont and secondary symbiont communities, all OTUs assigned to the reported symbionts of aphids were screened out from the OTU table, and the relative abundance of each symbiont was calculated.

All samples of *A. gossypii* were grouped according to geographic region and host plant (Table S2). First, we compared the alpha diversity indices of symbiont communities from different groups. Shannon and Simpson diversity indices for each group (sample size ≥ 3) were calculated with *vegan*. The alpha diversity data were found to deviate from normality (*P* < 0.05, Shapiro–Wilk test); therefore, we performed the non-parametric Kruskal–Wallis test to check for significant differences across all groups and conducted the non-parametric Wilcoxon tests for pairwise group comparisons.

Next, we investigated the patterns of beta diversity to address the relative importance of geography and host plant on symbiont and secondary symbiont communities. Beta diversity, i.e., the variation of symbiont community composition among differently grouped samples, was quantified using Bray–Curtis dissimilarity which considered the presence/absence and relative abundance of the individual symbiont. The Bray–Curtis distance was calculated between each pair of samples using the *vegdist* function in *vegan*. In the analyses of symbiont community, to reduce the influence of the most abundant *Buchnera*, the relative abundance data were logarithmically transformed with the *decostand* function of *vegan*. For the grouping scheme of geographic region, we assessed variation in community composition across all 23 groups, across ten groups with a sample size ≥ 3 (Zhejiang 1, 2, and 3 were treated as one group), and across three groups colonizing Rhamnaceae (sample size ≥ 3) (Beijing, Heilongjiang, and Liaoning 2). For the grouping scheme of host plant, we assessed variation in community composition among all 25 groups, among eleven groups with a sample size ≥ 3, among six groups with a sample size ≥ 5, and among eight groups from Beijing (sample size ≥ 3) (Asteraceae, Buxaceae, Crassulaceae, Cucurbitaceae, Lamiaceae, Malvaceae, Rhamnaceae, and Verbenaceae).

Principal component analysis (PCA) was firstly performed on the relative abundance matrix using the *prcomp* function in the R package *stats* to visualize variation among different groups in symbiont and secondary symbiont community compositions. PCA reduces the dimension of multivariate data and interprets such data diagrammatically. The resulting ordination was plotted with the R package *ggbiplot* [87]. Then, we used unconstrained and constrained ordination methods to visualize the Bray–Curtis dissimilarity. For the unconstrained ordination approach, we performed nonmetric multidimensional scaling (NMDS) on the Bray–Curtis distance matrix using the *metaMDS* function in *vegan* and presented two-dimensional plots by the R package *ggplot2* [88]. NMDS is found to always produce better ordinations than PCA [89]. For the constrained ordination approach, constrained principal coordinate analysis (cPCoA) was performed on the Bray–Curtis distance matrix using *capscale* and *anova.cca* functions in *vegan* and the resulting ordination was visualized by *ggplot2*. These ordination techniques are useful in representing community variation in response to environmental factors, such as geography and host plant in this study. In an ordination, samples that are close are more similar to one another than those that are far apart.

Based on the Bray–Curtis distance matrices, differences in symbiont and secondary symbiont community structures were also statistically analyzed with analysis of similarities (ANOSIM) and permutational multivariate analysis of variance (PERMANOVA). These two analyses are both resemblance-based permutation methods widely used in ecology and PERMANOVA is generally more powerful than ANOSIM to detect changes in community composition [90]. ANOSIM and PERMANOVA were applied using the *anosim* function and *adonis* function in *vegan*, respectively, and *P* values were obtained using 999 permutations. To further identify which symbionts were driving the differences in secondary symbiont community, we carried out the analysis of variance (ANOVA) tests in STAMP v2.1.3 [91] based on the relative abundances of each secondary symbiont from groups with a sample size ≥ 3. Pairwise group comparisons of the average relative abundances were then conducted using the post hoc Scheffé test, where the Bonferroni-adjusted *P* values were used to control the false discovery rate.

Furthermore, to test the effect of geographic distances among sampling sites in structuring the symbiont and secondary symbiont communities, the Pearson correlation coefficient between geographic distance matrix and Bray–Curtis distance matrix was calculated using Mantel test in *vegan*. The geographic distance matrix was generated using the Geographic Distance Matrix Generator v1.2.3 [92]. Mantel test allows to look for the correlation between two distance matrices. The null hypothesis that inter-point distances in these two matrices are not correlated was tested through a permutation procedure.

Finally, to explore potential interactions among different symbionts associated with *A. gossypii*, the Spearman correlation coefficients (*ρ*) between symbionts were calculated based on their relative abundances using the *cor* function in *stats* and were visualized in a heatmap with the R package *corrplot* [93].

## Results

### The Bacterial Diversity of *Aphis gossypii*

#### Overall Bacterial Diversity

**Fig. 1:**
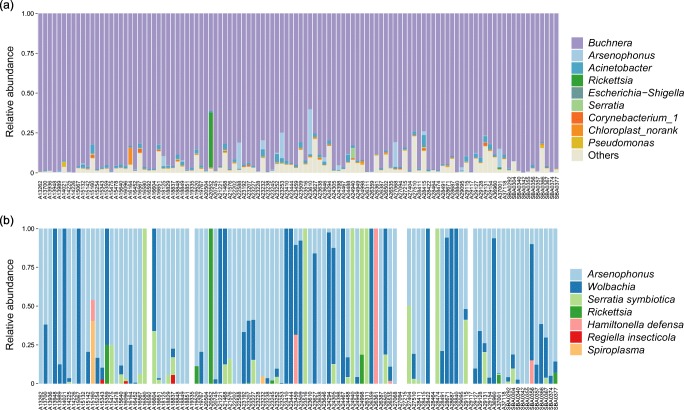
Barplots of bacterial communities (**a**) and secondary symbiont communities (**b**) associated with *Aphis gossypii* across all samples

After quality control, we obtained 3,867,639 16S rRNA gene sequences (35,160 reads per sample). A total of 1524 OTUs were identified at 97% similarity and were assigned into 39 phyla (Proteobacteria, 96.73% of total sequences), 104 classes (Gammaproteobacteria, 94.96%), 180 orders (Enterobacteriales, 93.48%), 310 families (Enterobacteriaceae, 93.48%), and 630 genera (Table S3). The bacterial community of *A. gossypii* was dominated by the primary endosymbiont *Buchnera aphidicola* (average relative abundance across all samples 91.79%), followed by the secondary symbiont *Arsenophonus* (1.11%) and the bacteria *Acinetobacter* (0.99%) (Fig. 1a, Table S3).

#### Symbiont Diversity

The alpha diversity of the symbiont community was very low (mean Shannon index = 0.063, mean Simpson index = 0.969) (Table S4). A total of eight aphid symbionts were detected in the cotton aphid. All samples harbored the primary endosymbiont *Buchnera aphidicola*. Along with *Arsenophonus*, they were also infected with *Rickettsia* (average relative abundance across all samples 0.32%), *Serratia symbiotica* (0.07%), *Wolbachia* (0.04%), *Hamiltonella defensa* (< 0.005%), *Regiella insecticola* (< 0.005%), and *Spiroplasma* (< 0.005%) (Fig. 1b, Table 1). Within the secondary symbiont community, the most prevalent bacteria were *Arsenophonus* (infection frequency 82/110), followed by *Wolbachia* (68/110), and *Serratia symbiotica* (32/110). *Hamiltonella defensa*, *Regiella insecticola*, and *Spiroplasma* were found to be low in both infection rate and abundance (Table 1). Many samples (69/110) were infected by at least two secondary symbionts in various combinations (Table 2). Co-infection with *Arsenophonus* and *Wolbachia* was the most common type (30/110), followed by multiple infections with *Arsenophonus*, *Serratia symbiotica*, and *Wolbachia* (13/110).

**Table 1:** Infection prevalence and average relative abundance of symbionts across all samples of *Aphis gossypii*

Symbiont	Infection frequency	Relative abundance (%)
*Buchnera aphidicola*	110/110	91.79
*Arsenophonus*	82/110	1.11
*Wolbachia*	68/110	0.04
*Serratia symbiotica*	32/110	0.07
*Rickettsia*	9/110	0.32
*Hamiltonella defensa*	6/110	< 0.005
*Regiella insecticola*	4/110	< 0.005
*Spiroplasma*	2/110	< 0.005

**Table 2:** Infection pattern of secondary symbionts within *Aphis gossypii*

Infection pattern	Secondary symbiont	Infection frequency
No infection		6/110
Single infection	*Arsenophonus*	19/110
	*Wolbachia*	11/110
	*Serratia symbiotica*	4/110
	*Hamiltonella defensa*	1/110
Co-infection	*Arsenophonus*–*Wolbachia*	30/110
	*Arsenophonus*–*Serratia*	6/110
	*Serratia*–*Wolbachia*	3/110
	*Arsenophonus*–*Rickettsia*	2/110
	*Arsenophonus*–*Spiroplasma*	1/110
	*Serratia*–*Rickettsia*	1/110
	*Wolbachia*–*Rickettsia*	1/110
Multiple infections	*Arsenophonus*–*Serratia*–*Wolbachia*	13/110
	*Arsenophonus*–*Hamiltonella*–*Wolbachia*	3/110
	*Arsenophonus*–*Rickettsia*–*Wolbachia*	2/110
	*Arsenophonus*–*Hamiltonella*–*Spiroplasma*	1/110
	*Arsenophonus*–*Regiella–Serratia*	1/110
	*Arsenophonus–Regiella*–*Wolbachia*	1/110
	*Serratia*–*Rickettsia*–*Wolbachia*	1/110
	*Arsenophonus*–*Regiella–Serratia*–*Wolbachia*	1/110
	*Arsenophonus*–*Hamiltonella*–*Rickettsia*–*Serratia*–*Wolbachia*	1/110
	*Arsenophonus*–*Regiella–Rickettsia*–*Serratia*–*Wolbachia*	1/110

### Symbiont Communities from Different Geographic Regions and Host Plants

#### Comparison of Alpha Diversity

No significant difference was detected among the alpha diversity indices of aphid symbionts from different geographic regions (*P* = 0.710 for Shannon index, *P* = 0.770 for Simpson index, Kruskal–Wallis test; *P* = 0.216–0.978 for Shannon index, *P* = 0.295–1.000 for Simpson index, Wilcoxon test). However, the Kruskal–Wallis test revealed statistical differences in the populations occupying different host plants (*P* = 0.006 < 0.01 for Shannon and Simpson indices). The symbionts within cotton aphid samples feeding on Buxaceae and Cucurbitaceae showed significantly higher and lower alpha diversities than samples on other plants, respectively (Buxaceae: *P* = 0.039 < 0.05 for Shannon index, *P* = 0.035 < 0.05 for Simpson index, Wilcoxon test; Cucurbitaceae: *P* = 0.002 < 0.01 for Shannon index, *P* = 0.015 < 0.05 for Simpson index, Wilcoxon test).

#### Pattern of Beta Diversity

**Fig. 2:**
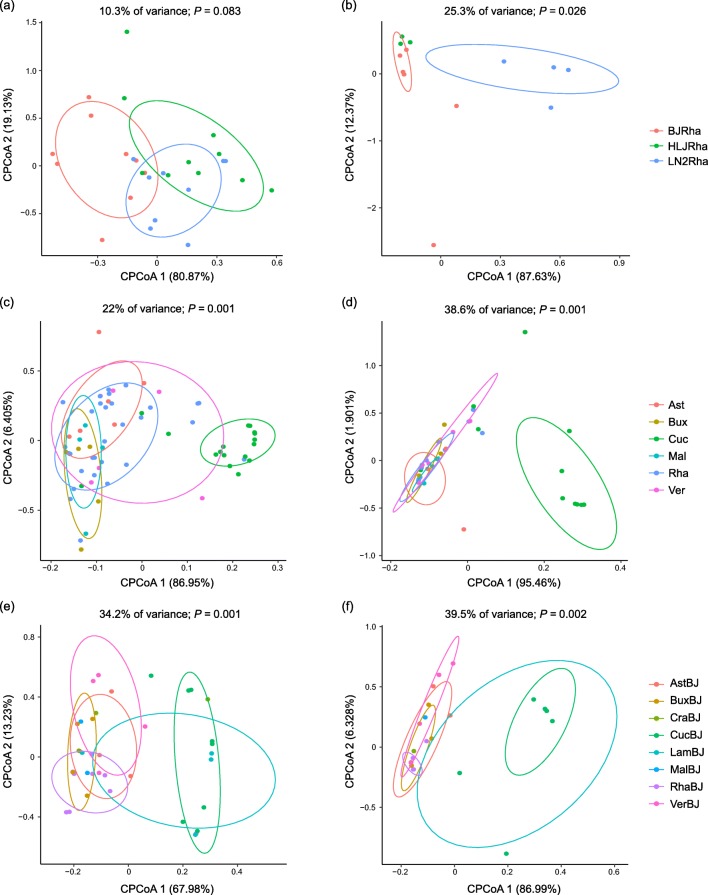
Constrained principal coordinate analyses (cPCoA) of Bray–Curtis distances of symbiont communities (**a**, **c**, **e**) and secondary symbiont communities (**b**, **d**, **f**) from three geographic groups feeding on Rhamnaceae (sample size ≥ 3) (**a**, **b**), six host plant groups with a sample size ≥5 (**c**, **d**), and eight host plant groups from Beijing (sample size ≥ 3) (**e**, **f**). See Table S2 for abbreviations

In the PCA, NMDS, and cPCoA analyses, no distinct clustering of symbiont composition for each geographic population was revealed (figures not shown), except for the cPCoA ordination of three groups feeding on Rhamnaceae (sample size ≥ 3), which showed that the samples from the same geographic region tended to cluster together and separate from others (Fig. 2a). ANOSIM tests found no significant differences across geographic regions (Table 3), whereas PERMANOVA tests detected statistical differences among 23 geographic populations (*R*^2^ = 0.265, *P* < 0.05) and across three groups feeding on Rhamnaceae (≥ 3 samples) (*R*^2^ = 0.170, *P* < 0.05). In the Mantel test, no significant correlation between Bray–Curtis dissimilarity and geographic distance could be observed (*r* = − 0.030, *P* = 0.681). For the symbiont communities associated with aphid populations occupying different host plants, although the PCA and NMDS ordinations did not show significant structuring patterns (figures not shown), both ANOSIM and PERMANOVA uncovered a strong effect of host plant on symbiont composition (*P* < 0.01, Table 3). Moreover, in the cPCoA analyses of six groups with a sample size ≥ 5 and eight groups from Beijing (sample size ≥ 3), the symbiont communities within aphids colonizing Cucurbitaceae tended to form a separate cluster (22–34.2% of variance, *P* = 0.001 < 0.01, Figs. 2c, e).

**Table 3:** ANOSIM and PERMANOVA results for symbiont and secondary symbiont communities from different groups

Group		Symbiont community		Secondary symbiont community	
		ANOSIM (*R*, *P*)	PERMANOVA (*R*^*2*^, *P*)	ANOSIM (*R*, *P*)	PERMANOVA (*R*^*2*^, *P*)
Geographic region	All 23 groups	0.053, 0.249	0.265, *0.049*	0.040, 0.223	0.256, 0.113
	10 groups (sample size ≥3)	0.002, 0.451	0.102, 0.386	−0.006, 0.487	0.132, 0.189
	3 groups (on Rhamnaceae, sample size ≥3)	0.091, 0.051	0.170, *0.034*	0.010, 0.324	0.099, 0.223
Host plant	All 25 groups	0.291, *0.001*	0.433, *0.001*	0.279, *0.002*	0.491, *0.001*
	11 groups (sample size ≥3)	0.258, *0.001*	0.319, *0.001*	0.280, *0.001*	0.429, *0.001*
	6 groups (sample size ≥5)	0.239, *0.001*	0.300, *0.001*	0.281, *0.001*	0.451, *0.001*
	8 groups (from Beijing, sample size ≥3)	0.278, *0.001*	0.433, *0.001*	0.304, *0.008*	0.367, *0.006*

Significant *P* values (*P* < 0.05) are in italics.

### Structural Variation in Secondary Symbiont Communities

#### Geographical Variation in Community Structure

**Fig. 3:**
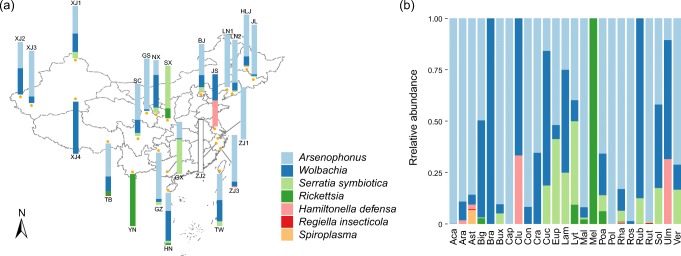
Barplots of secondary symbiont communities from different geographic regions (**a**) and host plants (**b**). The bars display relative abundances of distinct secondary symbionts. See Table S2 for abbreviations

The barplot of secondary symbiont compositions of different geographic populations is shown in Fig. 3a. No recognizable clustering was observed in the ordination analyses (figures not shown), except for the cPCoA of three groups feeding on Rhamnaceae (sample size ≥ 3) (25.3% of variance, *P* = 0.026 < 0.05, Fig. 2b). Neither ANOSIM nor PERMANOVA revealed significant differences in secondary symbiont community among geographic populations (Table 3). Mantel test also found no significant correlation between Bray–Curtis dissimilarity and geographic distance (*r* = 0.011, *P* = 0.413).

#### Community Variation with Respect to Host Plant

PCA did not exhibit good performance for the secondary symbionts from aphids exploiting different host plants (figures not shown). However, in the NMDS and cPCoA analyses of six groups with a sample size ≥ 5 and cPCoA of eight groups from Beijing (sample size ≥ 3), the communities within aphids feeding on Cucurbitaceae were clearly separated from other samples (cPCoA 38.6–39.5% of variance, *P* = 0.001–0.002 < 0.01, Figs. 2d, f). In addition, both ANOSIM and PERMANOVA revealed a significant effect of host plant on the secondary symbiont community (*P* < 0.01, Table 3). Among all detected secondary symbionts, the relative abundances of *Arsenophonus* and *Wolbachia* were found to significantly differ across different host plant groups (*P* < 0.01, ANOVA test, Figs. S1a, b). *Arsenophonus* was extremely dominant in the Acanthaceae, Caprifoliaceae, Polygonaceae, Rosaceae, and Rutaceae groups (average relative abundances across samples > 98%, Fig. 3b). However, it showed low abundance in the aphid samples feeding on Cucurbitaceae, Melastomataceae, and Ulmaceae (< 16%) and was not detected in the groups of Brassicaceae, Clusiaceae, and Rubiaceae (Fig. 3b). The post hoc Scheffé test also showed that the average relative abundance of *Arsenophonus* was significantly lower in the Cucurbitaceae group (*P* < 0.05, Fig. S1c). *Wolbachia* dominated in the communities from Brassicaceae, Clusiaceae, Cucurbitaceae, and Rubiaceae (> 65%, Fig. 3b). Its average relative abundance in the Cucurbitaceae group was significantly higher when tested using the post hoc Scheffé test (*P* < 0.05, Fig. S1d). However, *Wolbachia* accounted for much lower proportions (< 5%) in the communities from Acanthaceae, Asteraceae, Buxaceae, Melastomataceae, Rosaceae, and Rutaceae and was absent from the Caprifoliaceae and Polygonaceae groups (Fig. 3b).

### The Correlations Between Different Symbionts

**Fig. 4:**
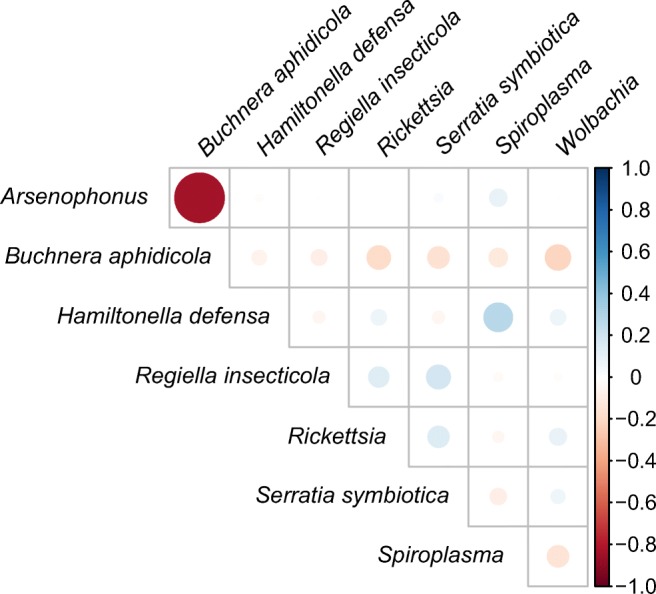
Heatmap of pairwise Spearman correlation coefficients of symbionts. Positive correlations are indicated as blue gradients from 0 to 1.0, and negative correlations are indicated as red gradients from 0 to − 1.0. The size of each circle is proportional to the significance level of the correlation coefficient

The Spearman correlation coefficients between symbionts were visualized as a heatmap (Fig. 4). *Buchnera aphidicola* seemed negatively correlated with secondary symbionts, particularly *Arsenophonus* (*ρ* = − 0.830, *P* < 0.01) and *Wolbachia* (*ρ* = − 0.211, *P* < 0.05) (Table S5). Both positive and negative correlations were observed between different secondary symbionts.

## Discussion

### Symbiont Diversity of *Aphis gossypii*

Our study revealed that the *A. gossypii* microbiota was dominated by a few bacterial taxa. Out of the top ten abundant genera, three were symbiotic bacteria, namely, *Buchnera*, *Arsenophonus*, and *Rickettsia* (Table S3). The third most abundant, *Acinetobacter*, has been reported in *A. gossypii* [73, 75, 76] and is common in insect gut communities [94, 95]. Many other bacteria detected here could be environmental or transient taxa.

As expected, *Buchnera aphidicola* was harbored by all aphid samples examined. It also showed the highest relative abundance in all samples. Considering the critical role of *Buchnera* in aphid survival and the long-term cospeciation of these two partners [8], the ubiquity and high abundance of *Buchnera* appear quite reasonable. Twelve OTUs belonging to *Buchnera* were identified. No phylogenetic concordance between *Buchnera* and their corresponding aphid hosts was found (data not shown). The presence of multiple phylotypes may therefore be correlated with mutation accumulation in the reduced *Buchnera* genomes, which seems to be caused by loss of DNA repair genes and fixation of slightly deleterious mutations through genetic drift [96, 97]. However, for phylotypes with extremely low abundance, the possibility that the mutations were artifacts introduced by PCR or sequencing errors cannot be ruled out.

Seven secondary symbionts were detected in this study, although their relative abundances were very low. *Spiroplasma*, which was not reported in *A. gossypii* previously, was identified in our sequencing data. *Spiroplasma* had extremely low relative abundance (< 0.005%) in two aphid samples, which may explain why it was not found before. The defensive symbiont *Hamiltonella defensa* was reported to infect all *A. gossypii* samples examined by Zhao et al. [74], Ayoubi et al. [77], and Zhang et al. [76]. However, in our study, it was just carried by six samples at very low abundance (< 0.005%). Our data showed that *Arsenophonus* was the predominant facultative symbiont, with the highest infection prevalence and abundance. It has been reported in previous studies of *A. gossypii* [66, 73–77]. Jousselin et al. [98] surveyed the distribution of *Arsenophonus* in aphids and revealed its high prevalence in the genus *Aphis*. Our results confirm their conclusion that *Arsenophonus* is a major bacterial partner of aphids. Most *Arsenophonus* in insects, including *A. gossypii*, was found to be associated with the lysogenic bacteriophage APSE [99], which is critical for *Hamiltonella* to confer protection against parasitoid wasps [100, 101]. In psyllids, the infection frequency of the APSE-bearing *Arsenophonus* has presented a positive correlation with parasitism, indicating a potential defensive role of *Arsenophonus* [102]. Therefore, we hypothesize that *Arsenophonus* in *A. gossypii* may play a similar role in providing resistance against parasitoids, especially in the case of rare *Hamiltonella* infection. Further experiments are required to determine the function of *Arsenophonus* in aphids.

### Impact of Geography and Host Plant on Symbiont Community

Geography has been reported to influence the microbial profiles of the Japanese, Australian, Hawaiian, and Chinese populations of *A. gossypii* feeding on a limited number of plant species [66, 73, 74]. However, in the present study, it contributed little to the cotton aphid’s symbiont community structure. No significant differences in symbiont or secondary symbiont communities over space were detected in the ordination analyses or statistical tests, except for three geographic groups colonizing the same plant family Rhamnaceae. This result is consistent with previous studies in which only a few plant species were included and suggests that when the host plant is not taken into account (i.e., the same or very few plant species), geography has an influence on the symbiont composition of aphids. In addition, Mantel tests detected no significant correlation between the geographic distances among sampling sites and Bray–Curtis dissimilarities of symbiont or secondary symbiont communities, which suggested negligible effect of spatial distance on the symbiont community structure.

Compared with the limited impact of geography, the host plant appeared to have played a greater role in shaping the symbiotic bacterial community associated with *A. gossypii*. The alpha diversity of symbionts was found to be significantly different across aphid populations exploiting different plants. ANOSIM and PERMANOVA tests also revealed a strong effect of the host plant on both symbiont and secondary symbiont communities. These findings are consistent with previous studies that showed that the populations of polyphagous aphids colonizing different plants tended to harbor different symbiont communities (e.g., *Acyrthosiphon pisum*, *Aphis craccivora*, *Aphis fabae*, and *Macrosiphum euphorbiae*) [54–56].

It is worth noting that the Cucurbitaceae-feeding cotton aphids hosted unique symbiont communities. They showed lower alpha diversity and were clustered together and separated from other samples in some ordination analyses. The post hoc Scheffé tests revealed significantly low-abundance *Arsenophonus* but high-abundance *Wolbachia* within the Cucurbitaceae-feeding populations. Correlations between certain endosymbionts and host plants have been repeatedly reported in polyphagous aphids, especially in the extensively studied pea aphid *Acyrthosiphon pisum* [35, 54, 55, 59, 60, 62]. For instance, the clover-adapted biotype of pea aphid was found to be associated with *Regiella insecticola* around the world [35, 38, 58, 65], and *Arsenophonus*-bearing locust populations were reported in *Aphis craccivora* [55, 62]. *A. gossypii* is a typical polyphagous species with a very wide range of host plants. Genetic differentiation has been found to occur among its host-associated populations [103–105]. Both host plant transfer experiments [106, 107] and molecular studies [104, 105, 108] have confirmed the existence of a cucurbits-specialized host race in *A. gossypii*. The special symbiont communities within cucurbits-feeding populations support the ecological specialization of *A. gossypii* on Cucurbitaceae from the perspective of symbiotic bacteria. However, it is not clear whether the associated symbionts have played a substantive role in host plant specialization of *A. gossypii*. Some studies suggested that facultative symbionts had an important influence on the host plant use of aphids [38–40]; some, however, doubted the direct impact of facultative symbionts on the plant adaptation of aphids [109, 110]. Further works based on a more extensive sampling are needed to present a comprehensive landscape of microbiota in Cucurbitaceae-feeding cotton aphids. Assessments of fitness effects by particular facultative symbionts are also necessary to elucidate the exact role of endosymbionts in host specialization.

### Symbiont–Symbiont Interactions

We conducted correlation analysis to assess the interactions between different symbionts. The correlation coefficients suggested antagonistic interactions between *Buchnera aphidicola* and secondary symbionts. In the pea aphid, *Serratia symbiotica* and *Rickettsia* have been reported to suppress the population density of *Buchnera* [111, 112]. Zhang et al. [76] also found a negative effect of *Hamiltonella* on the abundance of *Buchnera* in *A. gossypii*. These findings indicate competition between the primary and secondary symbionts for resources and survival niches within the same host aphid.

Multiple infections with secondary symbionts occurred commonly in our examined cotton aphids. Co-infections are often unstable [24, 113]. We hypothesize that such a high proportion of multiple infections may result from frequent horizontal transfers. *A. gossypii* is heteroecious holocyclic in China, alternating between primary host plants such as *Punica*, *Hibiscus*, and *Rhamnus* and various herbaceous secondary host plants [114]. The sexual phase [113] and migrations between different plants [115, 116] create opportunities for horizontal transfer of secondary symbionts among natural populations of *A. gossypii*. Co-infections may bring ecological benefits for the host aphids. *Acyrthosiphon pisum* co-infected with *Hamiltonella*–*Serratia* or *Hamiltonella*–*Fukatsuia* exhibited greater resistance to parasitoids [31, 32]. However, Polin et al. [117] reported that the *Acyrthosiphon pisum* strain co-infected with *Rickettsiella viridis* and *Hamiltonella defensa* was more exposed to ladybird predation than the singly *Rickettsiella*-infected strain. Ayoubi et al. [77] found that the *Hamiltonella*–*Arsenophonus* combination in *A. gossypii* conferred no resistance against parasitism by *Aphidius matricariae*. Therefore, the interactions between facultative symbionts seem very complicated, either synergistically or antagonistically, which was also indicated by the positive and negative Spearman correlation coefficients in our study.

## Conclusions

Based on an extensive sampling from different plants and regions in China, we analyzed the diversity of symbiotic bacteria within *Aphis gossypii* using Illumina sequencing of 16S rRNA gene. The microbiota of *A. gossypii* was dominated by heritable symbionts, among which *Buchnera aphidicola* and *Arsenophonus* were the predominant symbiont and facultative symbiont, respectively. The symbiont diversity was found to vary with the host plant rather than geography, suggesting an important role of the host plant in shaping the bacterial community structure. The cucurbits-adapted aphid populations harbored unique symbiont communities, which provide a good model to explore the direct or indirect impacts of facultative symbionts on host specialization. Moreover, the interactions between coexisting symbionts within *A. gossypii* were revealed to be very complicated.

## Electronic Supplementary Material

ESM 1 (DOCX 1.07 mb)
