# Supplementary material for: The Bacterial Flora Associated with the Polyphagous Aphid Aphis gossypii Glover (Hemiptera: Aphididae) Is Strongly Affected by Host Plants
Source: Microb Ecol. 2019 Dec 4;79(4):971–84. doi: 10.1007/s00248-019-01435-2 (PMC7198476; doi:10.1007/s00248-019-01435-2)
Supplement: Supplementary file 1 — (DOCX 1.07 mb) [file 248_2019_1435_MOESM1_ESM.docx]

**Supplementary material**

**Table S1.** Voucher information of aphid samples used in this study.

| Sample ID | Date | Host plant | Location |
| --- | --- | --- | --- |
| A13262 | 18 Apr. 2002 | *Rhamnus davurica* | Beijing, China |
| A13700 | 21 Aug. 2002 | *Lagenaria siceraria* var. *hispida* | Burqin, Xinjiang, China |
| A13936 | 20 Sep. 2002 | *Dendranthema morifolium* | Kashgar, Xinjiang, China |
| A13948 | 20 Sep. 2002 | *Lagenaria siceraria* | Kashgar, Xinjiang, China |
| A13989 | 24 Sep. 2002 | *Alcea rosea* | Pishan, Xinjiang, China |
| A14021 | 29 Sep. 2002 | Cucurbitaceae | Ruoqiang, Xinjiang, China |
| A14225 | 28 Apr. 2003 | *Rhamnus davurica* | Beijing, China |
| A14226 | 28 Apr. 2003 | *Zanthoxylum bungeanum* | Beijing, China |
| A15067 | 19 Aug. 2003 | *Cucurbita moschata* | Baoxing, Sichuan, China |
| A15132 | May 2004 | *Hibiscus syriacus* | Beijing, China |
| A15142 | 19 May 2004 | *Punica granatum* | Beijing, China |
| A15160 | 22 May 2004 | *Artemisia* sp. | Beijing, China |
| A15254 | 20 May 2004 | *Ipomoea nil* | Beijing, China |
| A15343 | 11 Aug. 2003 | *Chromolaena odorata* | Medog, Tibet, China |
| A15359 | 12 Aug. 2003 | *Zizania latifolia* | Medog, Tibet, China |
| A15475 | 28 Jul. 2004 | Poaceae | Tianquan, Sichuan, China |
| A15478 | 28 Jul. 2004 | Poaceae | Tianquan, Sichuan, China |
| A15640 | 24 Aug. 2004 | Poaceae | Zunyi, Guizhou, China |
| A16096 | 25 Apr. 2005 | *Zanthoxylum bungeanum* | Beijing, China |
| A16164 | 13 May 2005 | *Vitex negundo* var. *heterophylla* | Beijing, China |
| A16452 | 18 Jul. 2005 | *Buxus sinica* var. *parvifolia* | Bome, Tibet, China |
| A16567 | 08 May 2006 | *Rhamnus davurica* | Beijing, China |
| A16580 | 06 May 2006 | *Rhamnus globosa* | Beijing, China |
| A16592 | 25 Aug. 2005 | *Cucurbita moschata* | Burqin, Xinjiang, China |
| A16604 | 23 Aug. 2005 | *Luffa aegyptiaca* | Altay, Xinjiang, China |
| A16651 | 18 Aug. 2005 | *Cucurbita moschata* | Fuhai, Xinjiang, China |
| A18125 | 18 Nov. 2005 | *Dendranthema morifolium* | Beijing, China |
| A18829 | 15 May 2006 | *Rhamnus davurica* | Beijing, China |
| A18837 | 14 May 2006 | *Rhamnus davurica* | Beijing, China |
| A18848 | 10 May 2006 | *Rhamnus* sp. | Beijing, China |
| A18849 | 10 May 2006 | *Rhamnus globosa* | Beijing, China |
| A18851 | 10 May 2006 | *Hibiscus syriacus* | Beijing, China |
| A19335 | 09 Jul. 2006 | *Polygonum hydropiper* | Beijing, China |
| A19520 | 16 Nov. 2006 | *Hibiscus rosa-sinensis* | Ledong, Hainan, China |
| A19767 | 30 Jun. 2007 | *Rhamnus davurica* | Xiuyan, Liaoning, China |
| A20504 | 1 Oct. 2006 | *Cuscuta chinensis* | Panjin, Liaoning, China |
| A20552 | 28 Jul. 2007 | *Melastoma malabathricum* | Simao, Yunnan, China |
| A20745 | 21 Aug. 2007 | *Cotoneaster* sp. | Yuzhong, Gansu, China |
| A21221 | 9 Jul. 2008 | *Hypericum monogynum* | Jingyuan, Ningxia, China |
| A21468 | 28 Jun. 2008 | *Rubia cordifolia* | Jingyuan, Ningxia, China |
| A21576 | 2 Jul. 2008 | *Solanum tuberosum* | Mt. Liupan, Ningxia, China |
| A22202 | 21 Apr. 2009 | Rosaceae | Beijing, China |
| A22588 | 21 May 2009 | Rutaceae | Guza, Sichuan, China |
| A23192 | 22 May 2009 | *Rhamnus davurica* | Xiuyan, Liaoning, China |
| A23207 | 23 May 2009 | *Rhamnus davurica* | Xiuyan, Liaoning, China |
| A23227 | 23 May 2009 | *Rhamnus davurica* | Xiuyan, Liaoning, China |
| A23228 | 23 May 2009 | *Rhamnus davurica* | Xiuyan, Liaoning, China |
| A23232 | 25 May 2009 | *Rhamnus davurica* | Harbin, Heilongjiang, China |
| A23238 | 26 May 2009 | *Rhamnus davurica* | Harbin, Heilongjiang, China |
| A23245 | 26 May 2009 | *Rhamnus davurica* | Harbin, Heilongjiang, China |
| A23252 | 27 May 2009 | *Rhamnus davurica* | Dunhua, Jilin, China |
| A23253 | 27 May 2009 | *Rhamnus davurica* | Dunhua, Jilin, China |
| A23410 | 25 Jul. 2009 | Crassulaceae | Beijing, China |
| A23444 | 25 Jul. 2009 | *Cucurbita moschata* | Beijing, China |
| A23459 | 26 Jul. 2009 | *Ulmus pumila* | Beijing, China |
| A23509 | 26 Jul. 2009 | Cucurbitaceae | Beijing, China |
| A23516 | 26 Jul. 2009 | *Leonurus japonicus* | Beijing, China |
| A23610 | 15 Aug. 2009 | *Leonurus japonicus* | Beijing, China |
| A23627 | 15 Aug. 2009 | *Cucumis sativus* | Beijing, China |
| A23638 | 15 Aug. 2009 | *Cirsium japonicum* | Beijing, China |
| A23646 | 10 Sep. 2009 | Crassulaceae | Beijing, China |
| A24294 | 6 Apr. 2010 | *Cucurbita moschata* | Mt. Limu, Hainan, China |
| A24305 | 7 Apr. 2010 | *Cucumis sativus* | Mt. Limu, Hainan, China |
| A24398 | 14 May 2010 | *Rhamnus davurica* | Beijing, China |
| A24467 | 20 May 2010 | *Capsella bursa-pastoris* | Beijing, China |
| A24488 | 23 May 2010 | *Hibiscus syriacus* | Beijing, China |
| A24940 | 24 May 2011 | *Clerodendrum cyrtophyllum* | Nanning, Guangxi, China |
| A24949 | 24 May 2011 | *Vitex negundo* var. *cannabifolia* | Nanning, Guangxi, China |
| A24998 | 2 Jun. 2010 | *Punica granatum* | Xi’an, Shaanxi, China |
| A26311 | 23 Nov. 2010 | *Luffa acutangula* | Bobai, Guangxi, China |
| A26359 | 3 Apr. 2011 | *Hypericum monogynum* | Yangzhou, Jiangsu, China |
| A26361 | 3 Apr. 2011 | *Hypericum monogynum* | Yangzhou, Jiangsu, China |
| A26387 | 2 Jun. 2011 | Caprifoliaceae | Kaohsiung, Taiwan, China |
| A26502 | 15 Jun. 2011 | Solanaceae | Kaohsiung, Taiwan, China |
| A26535 | 10 Aug. 2011 | *Colocasia esculenta* | Wenzhou, Zhejiang, China |
| A27068 | 4 Aug. 2011 | *Justicia procumbens* | Hangzhou, Zhejiang, China |
| A27094 | 7 Aug. 2011 | *Luffa aegyptiaca* | Jinhua, Zhejiang, China |
| A27215 | 16 Aug. 2011 | *Clerodendrum* sp. | Nanning, Guangxi, China |
| A27404 | 24 Sep. 2011 | *Cucurbita moschata* | Beijing, China |
| A27510 | 13 May 2012 | *Buxus megistophylla* | Beijing, China |
| A28411 | 18 May 2013 | *Buxus megistophylla* | Beijing, China |
| A28415 | 19 May 2013 | *Fallopia multiflora* | Beijing, China |
| A28422 | 19 May 2013 | Lamiaceae | Beijing, China |
| A28464 | 10 Aug. 2013 | *Luffa aegyptiaca* | Beijing, China |
| A28474 | 13 Aug. 2013 | *Cucumis melo* | Beijing, China |
| A28491 | 13 Aug. 2013 | Asteraceae | Beijing, China |
| A28671 | 20 Jun. 2013 | *Campsis grandiflora* | Beijing, China |
| A28687 | 21 Jun. 2013 | *Scutellaria baicalensis* | Beijing, China |
| A28840 | 26 Oct. 2012 | Cucurbitaceae | Beijing, China |
| A29095 | 4 Jul. 2013 | *Buxus megistophylla* | Beijing, China |
| A29115 | 6 Aug. 2013 | *Acalypha australis* | Beijing, China |
| A29117 | 6 Aug. 2013 | *Trichosanthes kirilowii* | Beijing, China |
| A29127 | 6 Aug. 2013 | Verbenaceae | Beijing, China |
| A29128 | 6 Aug. 2013 | *Vitex negundo* | Beijing, China |
| A29131 | 7 Aug. 2013 | *Buxus megistophylla* | Beijing, China |
| A29154 | 9 Aug. 2013 | *Sedum* sp. | Beijing, China |
| A36960 | 22 Aug. 2016 | *Cucurbita moschata* | Beijing, China |
| A37001 | 20 May 2016 | *Campsis radicans* | Beijing, China |
| A37118 | 17 May 2016 | *Vitex negundo* var. *heterophylla* | Beijing, China |
| SBA0292 | 8 May 2008 | *Rhamnus davurica* | Xiuyan, Liaoning, China |
| SBA0304 | 8 May 2008 | *Rhamnus davurica* | Xiuyan, Liaoning, China |
| SBA0340 | 10 May 2008 | *Rhamnus davurica* | Xiuyan, Liaoning, China |
| SBA0342 | 10 May 2008 | *Rhamnus davurica* | Xiuyan, Liaoning, China |
| SBA0355 | 20 May 2008 | *Rhamnus ussuriensis* | Harbin, Heilongjiang, China |
| SBA0356 | 20 May 2008 | *Rhamnus ussuriensis* | Harbin, Heilongjiang, China |
| SBA0357 | 20 May 2008 | *Rhamnus ussuriensis* | Harbin, Heilongjiang, China |
| SBA0366 | 23 May 2008 | *Rhamnus ussuriensis* | Harbin, Heilongjiang, China |
| SBA0367 | 23 May 2008 | *Rhamnus ussuriensis* | Harbin, Heilongjiang, China |
| SBA0374 | 23 May 2008 | *Rhamnus ussuriensis* | Harbin, Heilongjiang, China |
| SBA0377 | 23 May 2008 | *Rhamnus ussuriensis* | Harbin, Heilongjiang, China |

**Table S2.** Grouping information of aphid samples used in this study.

| Geographic region (23 groups) | Number of samples | Sample ID |
| --- | --- | --- |
| Beijing (BJ) | 52 | A13262, A14225, A14226, A15132, A15142, A15160, A15254, A16096, A16164, A16567, A16580, A18125, A18829, A18837, A18848, A18849, A18851, A19335, A22202, A23410, A23444, A23459, A23509, A23516, A23610, A23627, A23638, A23646, A24398, A24467, A24488, A27404, A27510, A28411, A28415, A28422, A28464, A28474, A28491, A28671, A28687, A28840, A29095, A29115, A29117, A29127, A29128, A29131, A29154, A36960, A37001, A37118 |
| Gansu (GS) | 1 | A20745 |
| Guangxi (GX) | 4 | A24940, A24949, A26311, A27215 |
| Guizhou (GZ) | 1 | A15640 |
| Hainan (HN) | 3 | A19520, A24294, A24305 |
| Heilongjiang (HLJ) | 10 | A23232, A23238, A23245, SBA0355, SBA0356, SBA0357, SBA0366, SBA0367, SBA0374, SBA0377 |
| Jiangsu (JS) | 2 | A26359, A26361 |
| Jilin (JL) | 2 | A23252, A23253 |
| Liaoning1(LN1) | 1 | A20504 |
| Liaoning2 (LN2) | 9 | A19767, A23192, A23207, A23227, A23228, SBA0292, SBA0304, SBA0340, SBA0342 |
| Ningxia (NX) | 3 | A21221, A21468, A21576 |
| Shaanxi (SX) | 1 | A24998 |
| Sichuan (SC) | 4 | A15067, A15475, A15478, A22588 |
| Taiwan (TW) | 2 | A26387, A26502 |
| Tibet (TB) | 3 | A15343, A15359, A16452 |
| Xinjiang1 (XJ1) | 4 | A13700, A16592, A16604, A16651 |
| Xinjiang2 (XJ2) | 2 | A13936, A13948 |
| Xinjiang3 (XJ3) | 1 | A13989 |
| Xinjiang4 (XJ4) | 1 | A14021 |
| Yunnan (YN) | 1 | A20552 |
| Zhejiang1 (ZJ1) | 1 | A27068 |
| Zhejiang2 (ZJ2) | 1 | A27094 |
| Zhejiang3 (ZJ3) | 1 | A26535 |
| Host plant (25 groups) | Number of samples | Sample ID |
| Acanthaceae (Aca) | 1 | A27068 |
| Araceae (Ara) | 1 | A26535 |
| Asteraceae (Ast) | 6 | A13936, A15160, A15343, A18125, A23638, A28491 |
| Bignoniaceae (Big) | 2 | A28671, A37001 |
| Brassicaceae (Bra) | 1 | A24467 |
| Buxaceae (Bux) | 5 | A16452, A27510, A28411, A29095, A29131 |
| Caprifoliaceae (Cap) | 1 | A26387 |
| Clusiaceae (Clu) | 3 | A21221, A26359, A26361 |
| Convolvulaceae (Con) | 2 | A15254, A20504 |
| Crassulaceae (Cra) | 3 | A23410, A23646, A29154 |
| Cucurbitaceae (Cuc) | 20 | A13700, A13948, A14021, A15067, A16592, A16604, A16651, A23444, A23509, A23627, A24294, A24305, A26311, A27094, A27404, A28464, A28474, A28840, A29117, A36960 |
| Euphorbiaceae (Eup) | 1 | A29115 |
| Lamiaceae (Lam) | 4 | A23516, A23610, A28422, A28687 |
| Lythraceae (Lyt) | 2 | A15142, A24998 |
| Malvaceae (Mal) | 5 | A13989, A15132, A18851, A19520, A24488 |
| Melastomataceae (Mel) | 1 | A20552 |
| Poaceae (Poa) | 4 | A15359, A15475, A15478, A15640 |
| Polygonaceae (Pol) | 2 | A19335, A28415 |
| Rhamnaceae (Rha) | 30 | A13262, A14225, A16567, A16580, A18829, A18837, A18848, A18849, A19767, A23192, A23207, A23227, A23228, A23232, A23238, A23245, A23252, A23253, A24398, SBA0292, SBA0304, SBA0340, SBA0342, SBA0355, SBA0356, SBA0357, SBA0366, SBA0367, SBA0374, SBA0377 |
| Rosaceae (Ros) | 2 | A20745, A22202 |
| Rubiaceae (Rub) | 1 | A21468 |
| Rutaceae (Rut) | 3 | A14226, A16096, A22588 |
| Solanaceae (Sol) | 2 | A21576, A26502 |
| Ulmaceae (Ulm) | 1 | A23459 |
| Verbenaceae (Ver) | 7 | A16164, A24940, A24949, A27215, A29127, A29128, A37118 |

**Table S3.** Relative abundance of the top 10 bacterial phyla, classes, orders, families and genera in *Aphis gossypii*.

| Phylum | Class | Order | Family | Genus |
| --- | --- | --- | --- | --- |
| Proteobacteria/96.73% | Gammaproteobacteria/94.96% | Enterobacteriales/93.48% | Enterobacteriaceae/93.48% | ***Buchnera*/91.79%** |
| Actinobacteria/0.80% | Alphaproteobacteria/1.09% | Pseudomonadales/1.21% | Moraxellaceae/1.02% | ***Arsenophonus*/1.11%** |
| Firmicutes/0.77% | Actinobacteria/0.73% | Burkholderiales/0.52% | Rickettsiaceae/0.32% | *Acinetobacter*/0.99% |
| Bacteroidetes/0.72% | Betaproteobacteria/0.57% | Bacteroidales/0.38% | Corynebacteriaceae/0.21% | ***Rickettsia*/0.32%** |
| Cyanobacteria/0.20% | Bacilli/0.42% | Rickettsiales/0.37% | Oxalobacteraceae/0.20% | *Escherichia–Shigella*/0.32% |
| Thermotogae/0.15% | Bacteroidia/0.38% | Clostridiales/0.29% | Chloroplast_s_norank/0.19% | ***Serratia*/0.22%** |
| Deinococcus–Thermus/0.13% | Clostridia/0.29% | Bacillales/0.26% | Comamonadaceae/0.19% | *Corynebacterium_1*/0.20% |
| Chloroflexi/0.11% | Chloroplast/0.19% | Micrococcales/0.25% | Pseudomonadaceae/0.18% | *Chloroplast_norank*/0.19% |
| Nitrospirae/0.08% | Thermotogae/0.15% | Rhizobiales/0.25% | Ruminococcaceae/0.16% | *Pseudomonas*/0.18% |
| Acidobacteria/0.06% | Deltaproteobacteria/0.10% | Corynebacteriales/0.24% | Halomonadaceae/0.20% | *Halomonas*/0.16% |

Symbionts are indicated in bold. *Serratia* includes the endosymbiont *Serratia symbiotica* and free-living species.

**Table S4.** Alpha diversity of bacterial and symbiont communities for each sample.

| Sample ID | Bacterial community | | | Symbiont community | | |
| --- | --- | --- | --- | --- | --- | --- |
|  | Number of OTUs | Shannon | Simpson | Number of OTUs | Shannon | Simpson |
| A13262 | 112 | 0.115 | 0.975 | 4 | 0.014 | 0.997 |
| A13700 | 112 | 0.135 | 0.970 | 6 | 0.010 | 0.998 |
| A13936 | 219 | 0.157 | 0.968 | 4 | 0.020 | 0.995 |
| A13948 | 118 | 0.095 | 0.980 | 5 | 0.016 | 0.996 |
| A13989 | 84 | 0.123 | 0.972 | 8 | 0.018 | 0.996 |
| A14021 | 108 | 0.473 | 0.853 | 5 | 0.007 | 0.999 |
| A14036 | 696 | 3.227 | 0.305 | 6 | 0.022 | 0.994 |
| A14225 | 106 | 0.079 | 0.983 | 5 | 0.020 | 0.995 |
| A14226 | 53 | 0.046 | 0.990 | 5 | 0.007 | 0.999 |
| A15067 | 214 | 0.343 | 0.919 | 6 | 0.048 | 0.984 |
| A15132 | 166 | 0.437 | 0.884 | 7 | 0.017 | 0.996 |
| A15142 | 229 | 0.330 | 0.923 | 8 | 0.017 | 0.996 |
| A15160 | 262 | 1.166 | 0.678 | 6 | 0.008 | 0.998 |
| A15254 | 160 | 0.209 | 0.952 | 7 | 0.014 | 0.997 |
| A15343 | 203 | 0.308 | 0.929 | 5 | 0.006 | 0.999 |
| A15359 | 198 | 0.383 | 0.905 | 7 | 0.040 | 0.989 |
| A15475 | 79 | 0.115 | 0.973 | 6 | 0.016 | 0.996 |
| A15478 | 200 | 0.369 | 0.911 | 7 | 0.016 | 0.996 |
| A15640 | 204 | 0.417 | 0.896 | 7 | 0.020 | 0.995 |
| A16096 | 263 | 0.370 | 0.914 | 7 | 0.017 | 0.996 |
| A16112 | 538 | 3.637 | 0.102 | 8 | 0.048 | 0.987 |
| A16164 | 250 | 0.862 | 0.698 | 6 | 0.028 | 0.993 |
| A16452 | 139 | 0.200 | 0.952 | 6 | 0.005 | 0.999 |
| A16567 | 340 | 1.308 | 0.663 | 5 | 0.007 | 0.998 |
| A16580 | 181 | 0.301 | 0.927 | 7 | 0.007 | 0.998 |
| A16592 | 174 | 0.208 | 0.953 | 9 | 0.028 | 0.992 |
| A16604 | 144 | 0.230 | 0.946 | 7 | 0.250 | 0.875 |
| A16651 | 291 | 1.087 | 0.680 | 7 | 0.031 | 0.991 |
| A18125 | 186 | 0.568 | 0.803 | 9 | 0.013 | 0.997 |
| A18829 | 194 | 0.375 | 0.907 | 5 | 0.013 | 0.997 |
| A18837 | 248 | 0.370 | 0.916 | 5 | 0.057 | 0.980 |
| A18848 | 292 | 0.859 | 0.783 | 7 | 0.027 | 0.993 |
| A18849 | 224 | 0.582 | 0.849 | 5 | 0.004 | 0.999 |
| A18851 | 182 | 0.208 | 0.953 | 5 | 0.019 | 0.995 |
| A19335 | 156 | 0.242 | 0.940 | 5 | 0.021 | 0.995 |
| A19520 | 315 | 1.008 | 0.727 | 6 | 0.025 | 0.993 |
| A19767 | 189 | 0.381 | 0.906 | 10 | 0.661 | 0.538 |
| A20504 | 130 | 0.165 | 0.962 | 5 | 0.019 | 0.995 |
| A20552 | 237 | 0.995 | 0.491 | 6 | 0.018 | 0.996 |
| A20745 | 180 | 0.157 | 0.967 | 6 | 0.159 | 0.936 |
| A21221 | 226 | 0.371 | 0.915 | 8 | 0.023 | 0.995 |
| A21468 | 263 | 1.212 | 0.671 | 4 | 0.011 | 0.997 |
| A21576 | 113 | 0.229 | 0.943 | 6 | 0.467 | 0.716 |
| A22202 | 288 | 0.677 | 0.835 | 5 | 0.006 | 0.999 |
| A22588 | 204 | 0.679 | 0.680 | 9 | 0.042 | 0.989 |
| A23192 | 291 | 0.447 | 0.899 | 8 | 0.014 | 0.997 |
| A23207 | 197 | 0.519 | 0.869 | 5 | 0.006 | 0.999 |
| A23227 | 132 | 0.288 | 0.917 | 7 | 0.275 | 0.868 |
| A23228 | 82 | 0.118 | 0.973 | 6 | 0.011 | 0.997 |
| A23232 | 171 | 1.163 | 0.636 | 7 | 0.041 | 0.989 |
| A23238 | 87 | 0.077 | 0.984 | 6 | 0.073 | 0.977 |
| A23245 | 223 | 0.203 | 0.956 | 7 | 0.493 | 0.703 |
| A23252 | 146 | 0.823 | 0.762 | 5 | 0.010 | 0.998 |
| A23253 | 152 | 1.090 | 0.581 | 5 | 0.004 | 0.999 |
| A23410 | 181 | 0.580 | 0.856 | 9 | 0.012 | 0.997 |
| A23444 | 270 | 0.512 | 0.879 | 6 | 0.011 | 0.997 |
| A23459 | 173 | 0.396 | 0.901 | 6 | 0.004 | 0.999 |
| A23509 | 328 | 1.362 | 0.654 | 5 | 0.631 | 0.564 |
| A23516 | 283 | 0.607 | 0.852 | 5 | 0.015 | 0.996 |
| A23610 | 476 | 1.511 | 0.441 | 5 | 0.008 | 0.998 |
| A23627 | 508 | 1.734 | 0.568 | 6 | 0.142 | 0.940 |
| A23638 | 370 | 0.908 | 0.785 | 5 | 0.016 | 0.996 |
| A23640 | 690 | 3.858 | 0.205 | 8 | 0.005 | 0.999 |
| A23646 | 143 | 0.626 | 0.813 | 6 | 0.142 | 0.940 |
| A24294 | 349 | 1.433 | 0.636 | 7 | 0.035 | 0.991 |
| A24305 | 150 | 0.188 | 0.958 | 7 | 0.041 | 0.988 |
| A24398 | 175 | 0.305 | 0.907 | 7 | 0.263 | 0.866 |
| A24467 | 178 | 0.306 | 0.928 | 7 | 0.009 | 0.998 |
| A24488 | 168 | 0.772 | 0.781 | 6 | 0.007 | 0.999 |
| A24940 | 428 | 1.095 | 0.690 | 6 | 0.006 | 0.999 |
| A24949 | 364 | 0.646 | 0.849 | 4 | 0.005 | 0.999 |
| A24998 | 320 | 0.648 | 0.842 | 5 | 0.027 | 0.993 |
| A26126 | 769 | 3.172 | 0.326 | 6 | 0.008 | 0.998 |
| A26311 | 95 | 0.104 | 0.977 | 7 | 0.007 | 0.999 |
| A26359 | 112 | 0.102 | 0.978 | 7 | 0.019 | 0.995 |
| A26361 | 595 | 1.015 | 0.775 | 7 | 0.450 | 0.727 |
| A26387 | 152 | 0.254 | 0.940 | 4 | 0.005 | 0.999 |
| A26502 | 377 | 1.023 | 0.741 | 4 | 0.005 | 0.999 |
| A26535 | 338 | 0.337 | 0.930 | 6 | 0.006 | 0.999 |
| A27068 | 163 | 0.730 | 0.674 | 8 | 0.020 | 0.995 |
| A27094 | 152 | 0.302 | 0.927 | 6 | 0.016 | 0.996 |
| A27215 | 521 | 1.261 | 0.708 | 5 | 0.148 | 0.936 |
| A27404 | 83 | 0.120 | 0.971 | 5 | 0.005 | 0.999 |
| A27510 | 359 | 1.817 | 0.548 | 4 | 0.007 | 0.999 |
| A28411 | 122 | 0.204 | 0.951 | 6 | 0.007 | 0.999 |
| A28413 | 361 | 2.737 | 0.319 | 7 | 0.016 | 0.996 |
| A28415 | 191 | 1.507 | 0.550 | 8 | 0.038 | 0.990 |
| A28422 | 88 | 0.162 | 0.959 | 5 | 0.009 | 0.998 |
| A28464 | 233 | 0.218 | 0.954 | 6 | 0.006 | 0.999 |
| A28474 | 106 | 0.132 | 0.970 | 7 | 0.107 | 0.958 |
| A28491 | 263 | 0.449 | 0.891 | 9 | 0.036 | 0.991 |
| A28671 | 222 | 0.454 | 0.890 | 4 | 0.007 | 0.999 |
| A28687 | 532 | 0.917 | 0.796 | 7 | 0.042 | 0.988 |
| A28840 | 81 | 0.064 | 0.987 | 7 | 0.034 | 0.991 |
| A29095 | 114 | 0.231 | 0.931 | 8 | 0.617 | 0.674 |
| A29115 | 238 | 0.762 | 0.799 | 6 | 0.025 | 0.993 |
| A29117 | 60 | 0.068 | 0.985 | 9 | 0.022 | 0.994 |
| A29127 | 237 | 0.857 | 0.766 | 6 | 0.010 | 0.998 |
| A29128 | 236 | 0.715 | 0.812 | 7 | 0.014 | 0.997 |
| A29131 | 462 | 2.102 | 0.403 | 6 | 0.012 | 0.997 |
| A29154 | 263 | 1.238 | 0.669 | 6 | 0.008 | 0.998 |
| A36960 | 525 | 1.009 | 0.776 | 4 | 0.014 | 0.997 |
| A37001 | 301 | 0.696 | 0.726 | 6 | 0.010 | 0.998 |
| A37118 | 358 | 0.885 | 0.785 | 4 | 0.020 | 0.995 |
| SBA0292 | 158 | 0.232 | 0.948 | 8 | 0.018 | 0.996 |
| SBA0304 | 587 | 0.714 | 0.852 | 6 | 0.018 | 0.996 |
| SBA0340 | 141 | 0.167 | 0.964 | 7 | 0.010 | 0.998 |
| SBA0342 | 30 | 0.024 | 0.995 | 7 | 0.018 | 0.996 |
| SBA0355 | 121 | 0.123 | 0.974 | 8 | 0.040 | 0.989 |
| SBA0356 | 136 | 0.383 | 0.902 | 13 | 0.457 | 0.768 |
| SBA0357 | 132 | 0.121 | 0.974 | 6 | 0.014 | 0.997 |
| SBA0366 | 245 | 1.141 | 0.656 | 7 | 0.015 | 0.996 |
| SBA0367 | 216 | 0.389 | 0.909 | 6 | 0.011 | 0.997 |
| SBA0374 | 106 | 0.087 | 0.982 | 5 | 0.016 | 0.996 |
| SBA0377 | 282 | 0.297 | 0.937 | 8 | 0.018 | 0.996 |
| Mean | 238 | 0.673 | 0.823 | 6 | 0.063 | 0.969 |

**Table S5.** Spearman correlation coefficients of symbionts in *Aphis gossypii.*

| Symbiont 1 | Symbiont 2 | Correlation coefficient | *P* value |
| --- | --- | --- | --- |
| *Arsenophonus* | *Buchnera aphidicola* | **–0.830**^**^ | 0 |
| *Arsenophonus* | *Hamiltonella defensa* | –0.020 | 0.837 |
| *Arsenophonus* | *Regiella insecticola* | 0.007 | 0.945 |
| *Arsenophonus* | *Rickettsia* | 0 | 1.000 |
| *Arsenophonus* | *Serratia symbiotica* | 0.025 | 0.793 |
| *Arsenophonus* | *Spiroplasma* | 0.098 | 0.307 |
| *Arsenophonus* | *Wolbachia* | 0.004 | 0.963 |
| *Buchnera aphidicola* | *Hamiltonella defensa* | –0.070 | 0.469 |
| *Buchnera aphidicola* | *Regiella insecticola* | –0.081 | 0.400 |
| *Buchnera aphidicola* | *Rickettsia* | –0.181 | 0.058 |
| *Buchnera aphidicola* | *Serratia symbiotica* | –0.152 | 0.113 |
| *Buchnera aphidicola* | *Spiroplasma* | –0.112 | 0.243 |
| *Buchnera aphidicola* | *Wolbachia* | **–0.211**^*^ | 0.027 |
| *Hamiltonella defensa* | *Regiella insecticola* | –0.047 | 0.629 |
| *Hamiltonella defensa* | *Rickettsia* | 0.078 | 0.418 |
| *Hamiltonella defensa* | *Serratia symbiotica* | –0.049 | 0.610 |
| *Hamiltonella defensa* | *Spiroplasma* | **0.272**^**^ | 0.004 |
| *Hamiltonella defensa* | *Wolbachia* | 0.077 | 0.423 |
| *Regiella insecticola* | *Rickettsia* | 0.134 | 0.164 |
| *Regiella insecticola* | *Serratia symbiotica* | 0.186 | 0.051 |
| *Regiella insecticola* | *Spiroplasma* | –0.026 | 0.784 |
| *Regiella insecticola* | *Wolbachia* | –0.020 | 0.837 |
| *Rickettsia* | *Serratia symbiotica* | 0.148 | 0.122 |
| *Rickettsia* | *Spiroplasma* | –0.041 | 0.674 |
| *Rickettsia* | *Wolbachia* | 0.094 | 0.331 |
| *Serratia symbiotica* | *Spiroplasma* | –0.085 | 0.375 |
| *Serratia symbiotica* | *Wolbachia* | 0.063 | 0.510 |
| *Spiroplasma* | *Wolbachia* | –0.150 | 0.118 |

^*^*P* < 0.05, ^**^*P* < 0.01.


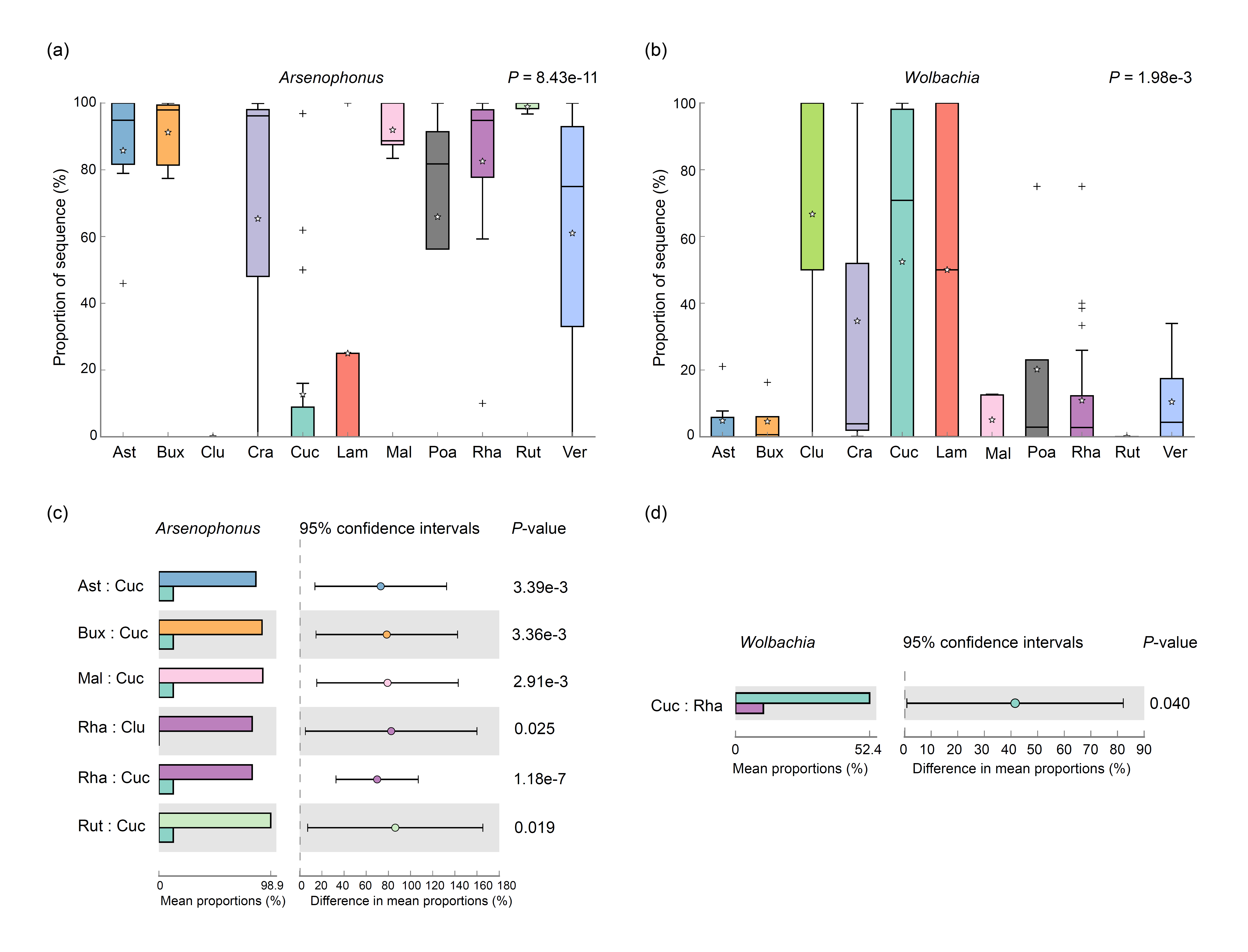


**Fig. S1** Boxplots of the relative abundances of *Arsenophonus* (**a**) and *Wolbachia* (**b**) associated with aphid samples feeding on different plants (sample size ≥ 3). The pairwise post hoc Scheffé test (*P* < 0.05) for the average relative abundances of *Arsenophonus* (**c**) and *Wolbachia* (**d**) associated with aphid samples feeding on different plants (sample size ≥ 3). See Table S2 for abbreviations.
